# Biogenic Silver Nanoparticles for Trace Colorimetric Sensing of Enzyme Disrupter Fungicide Vinclozolin

**DOI:** 10.3390/nano9111604

**Published:** 2019-11-12

**Authors:** Masood Hussain, Ayman Nafady, Sirajuddin, Ahmet Avcı, Erol Pehlivan, Jan Nisar, Syed Tufail Hussain Sherazi, Aamna Balouch, Muhammad Raza Shah, Omar A. Almaghrabi, Muhammad Anwar Ul-Haq

**Affiliations:** 1 National Centre of Excellence in Analytical Chemistry, University of Sindh, Jamshoro 76080, Pakistan; masood.hussain59@yahoo.com (M.H.); tufail.sherazi@gmail.com (S.T.H.S.); aamna_balouch@yahoo.com (A.B.); 2 Department of Chemistry, College of Science, King Saud University, Riyadh 11451, Saudi Arabia; 3 Chemistry Department, Faculty of Science, Sohag University, Sohag 82524, Egypt; 4 HEJ Research Institute of Chemistry, International Center for Chemical and Biological Center, University of Karachi, Karachi 75270, Pakistan; raza.shah@iccs.edu (M.R.S.); anwarulhaq196@gmail.com (M.A.U.-H.); 5 Department of Biomedical Engineering, Necmettin Erbakan University, Konya 42060, Turkey; ahmetavci@erbakan.edu.tr; 6 Department of Chemical Engineering, Selcuk University, Konya 42079, Turkey; erolpehlivan@gmail.com; 7 National Centre of Excellence in Physical Chemistry, University of Peshawar, Peshawar 25120, Pakistan; pashkalawati@gmail.com; 8 Department of Biological Sciences, Faculty of Science, University of Jeddah, Jeddah 21959, Saudi Arabia; oalmaghrabi@uj.edu.sa

**Keywords:** clove extract, silver nanoparticles, colorimetric sensor, vinclozolin, real water samples

## Abstract

We report a novel, simple, efficient, and green protocol for biogenic synthesis of silver nanoparticles (AgNPs) in aqueous solution using clove (*Syzygium aromaticum*) extract as a reducing and protecting agent. Ultraviolet-visible (UV-Vis) spectroscopy was employed to monitor the localized surface plasmon resonance (LSPR) band of clove extract-derived AgNPs prepared under various conditions. Fourier-transform infrared (FTIR) spectroscopy analysis provided information about the surface interaction of the clove extract with the AgNPs. Ultrahigh-resolution transmission electron microscopy (UHRTEM) results confirmed the formation of spherical, uniformly distributed clove extract-capped AgNPs with sizes in the range of 2–20 nm (average size: 14.4 ± 2 nm). Powder X-ray diffractometry analysis (PXRD) illustrated the formation of pure crystalline AgNPs. These AgNPs were tested as a colorimetric sensor to detect trace amounts of vinclozolin (VIN) by UV-Vis spectroscopy for the first time. The AgNP-based sensor demonstrated very sensitive and selective colorimetric detection of VIN, in the range of 2–16 µM (*R*^2^ = 0.997). The developed sensor was green, simple, sensitive, selective, economical, and novel, and could detect trace amounts of VIN with limit of detection (LOD) = 21 nM. Importantly, the sensor was successfully employed for the determination of VIN in real water samples collected from various areas in Turkey.

## 1. Introduction

**Scheme 1:**
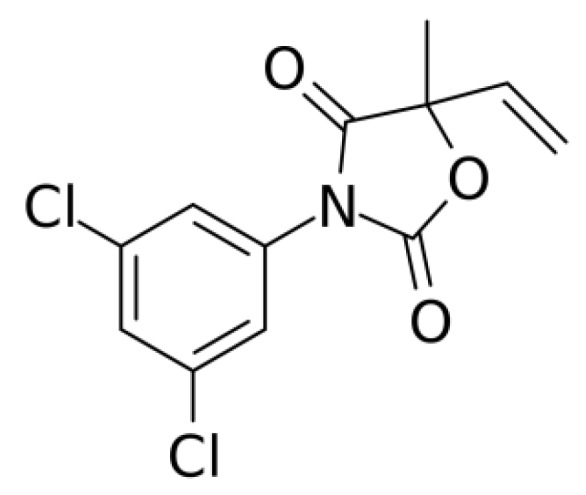
Vinclozolin (VIN): 3-(3,5-dichlorophenyl)-5-ethenyl-5-methyl-2,4-oxazolidinedione.

Vinclozolin (VIN) is a dicarboximide fungicide (Scheme 1) commonly used to control various diseases in vegetables and fruits. VIN is mostly applied to save fruits, crops, and vegetables from *Botrytis cinerea* and *Sclerotinia sclerotiorum* fungi [1,2]. It is a hazardous and carcinogenic pesticide that can influence our environment and affect animal and bird life. Because VIN easily damages the endocrine system and causes anti-androgenic effects [3,4], its determination and selective detection is crucial for environmental protection and securing the community from its carcinogenic effect.

Various methods such as gas chromatography tandem mass spectroscopy (GC–MS/MS) [5], high-performance liquid chromatography (HPLC) [6], solid phase micro-extraction coupled to GC with an electron capture detector (SPME–GC–ECD) [7], SPME coupled to high performance liquid chromatography (SPME–HPLC) [8], HPLC followed by GC–MS/MS [9], and electrochemical methods [10] have been reported in the literature for the detection of VIN. However, these methods use various volatile and hazardous solvents and chemicals, which are toxic to humans, aquatic life, and the environment. Moreover, although some of these methods are highly sensitive, they need complicated protocols for sample preparation and processing, and their running cost is high, making them unfit for routine analysis.

Metal nanoparticles are receiving considerable attention because of their novel applications in several fields such as catalysis, sensors, optical devices, electronics, and antimicrobial applications [11,12,13]. In particular, because of their unique chemical and physical properties, they are used for the fabrication of high-selectivity sensors to detect pesticides. Several sensors such as electrochemical sensors, colorimetric sensors, and amperometric biosensors have been used for the detection of various pesticides in different samples [14,15,16]. Moreover, the use of metal nanoparticles as colorimetric probes is receiving wide interest owing to advantages such as simplicity, economic viability, and fast response. Ag, Cu, and Au nanoparticles (NPs) are particularly attractive because they exhibit a unique and prominent property: localized surface plasmon resonance (LSPR) and high extinction coefficients [17,18]. A prominent LSPR band is generated when the conduction band electrons and electrons of the outer surface mutually oscillate after the particle size reaches the nanoscale. Small changes occurring on the surface of metal nanoparticles can cause a variation in the solution color, resulting in an LSPR absorption band in the UV-Vis spectra. Ag, Au, and Cu nanoparticles with sizes in the range of 5–70 nm exhibit bands at approximately 400 nm (yellow), 520 nm (light red), and 570 nm (red), respectively [19,20,21,22] and have been used as sensing probes to detect various analytes, including pesticides.

Among metal nanoparticles, Ag nanoparticles (AgNPs) have received considerable attention because of their surface plasmon properties, ease of preparation, green nature, antimicrobial properties, and affordable cost. Several chemical synthetic protocols [23,24,25] as well as biosynthetic procedures [26] and various reagents such as polymers, citrate, surfactants, and plant extracts have been used as reducing and/or stabilizing agents to fabricate AgNPs. Compared to chemical reduction methods, biosynthetic procedures are considered greener and are more preferred because in these processes, chemicals such as sodium borohydride, hydrazine, surfactants, and polymers, which may have carcinogenic or other toxic effects, are not used as reducing and protecting agents. Shrivas et al. [27] have developed a colorimetric probe based on AgNPs for sensing Endrin pesticide in water and food samples. Moreover, a spectrophotometric method has been reported for the detection of Dipterex using citrate-capped AgNPs [28].

Here, we report a new LSPR colorimetric sensor based on AgNPs, biologically synthesized from clove extract, to determine VIN in water samples. For the first time, these biologically synthesized AgNPs were used as a colorimetric sensor for low-level detection of VIN in real water samples. To the best of our knowledge, no UV-Vis spectrometric method has been reported for the detection of VIN. The developed sensor is economical and green, and it can easily detect low levels of the endocrine disruptor VIN.

## 2. Experimental

### 2.1. Materials and Reagents

Highly pure analytical grade chemicals and reagents utilized in this study such as AgNO_3_ (99.9%), HCl (37%), NaOH (98%) as well as CrCl_3_·6H_2_O, Ni(NO_3_)_2_·6H_2_O, and Co(NO_3_)_2_·6H_2_O were purchased from Merck (Darmstadt Germany) and Fluka Chemicals (Buchs, Switzerland). Other reagents such as vinclozolin (VIN), pymetrozine (PY), parathion-ethyl (PE), procymidone (PR), haloxyfop-p-methyl (HpM), lambda cyhalothrin (LC), and azinphos-ethyl (AE) were obtained from Sigma-Aldrich (Darmstadt, Germany). The stock solutions were prepared in Milli-Q water with certain quantities (per mg) of the chemicals. All experiments were carried out at ambient temperature (25 °C).

### 2.2. Instrumentation

A UV-Vis spectrophotometer (U-3900, 2J2-0013, Hitachi, Tokyo, Japan) was used to record the LSPR band of clove extract-coated AgNPs under specific conditions and colorimetric studies were performed in the range of 200–800 nm. The surface interactions of AgNPs with clove extract were examined using a Fourier-transform infrared (FTIR) spectrophotometer (Bruker Vertex-70, Ettlingen, Germany). Powder X-ray diffraction (PXRD) study was performed with JES-FA/300 JEOL, Tokyo, (Japan) using the Cu Kα (λ = 1.54060 Å) radiation at 40 kV and 30 mA, and a scanning rate of 2°/min to check the crystalline nature of AgNPs. Solid samples were obtained by heating the solutions in a water bath till all water was completely evaporated. Ultrahigh-resolution transmission electron microscopy (UHRTEM) images were recorded with JEOL JEM–2100 (Tokyo, Japan) at 300 kV to study the size distribution and morphology of the clove-AgNP-based colorimetric sensor before and after sensing of VIN. Various high-resolution photographs of the clove-AgNPs were recorded to confirm parameter optimization and study the colorimetric behavior of the sensor in the presence of VIN.

### 2.3. Synthesis of Clove-AgNPs

Firstly, cloves were ground into a powder with a mortar and pestle. Then, clove extract was prepared by dissolving 20 g of the powder in 1000 mL of deionized water for 36 h and filtering the solution; the solid residue on the filter paper was discarded. Clove-AgNPs were synthesized by using the prepared clove extract as a reducing agent as follows: 0.1 mL of 0.1 M AgNO_3_ solution was diluted to 8.0 mL by adding Milli-Q water in a beaker. To this, 0.1 mL of clove extract was added, and the final solution was made up to 10 mL with Milli-Q water. Subsequently, the solution was constantly stirred with a magnetic bead at room temperature for 20 min. Then, the solution was left undisturbed for 10 min for the complete formation of AgNPs; the solution turned yellow, indicating the formation of AgNPs. Small nanoparticles were formed under a neutral pH. UV-Vis spectra of the prepared AgNPs were recorded.

### 2.4. Colorimetric Sensing of VIN with Clove-AgNPs

Colorimetric recognition of VIN was conducted at room temperature. Different volumes (20, 40, 60, 80, 100, 120, 140, and 160 µL) of 0.001 M VIN stock solution were mixed with 3 mL of the biosynthesized AgNP solution and diluted to the final volume of 10 mL with Milli-Q water. Each working solution was left undisturbed for 40 min and then a sufficient volume was poured into a 1 cm quartz cell to study the colorimetric response. Spectroscopic study was conducted in the wavelength range from 200 nm to 800 nm against Milli-Q water as a blank reagent. The color change from yellowish to dark brown was considered the visual output and the change in absorbance (∆ absorbance) during the LSPR study was determined as a measure of colorimetric sensing of VIN. The respective color changes were recorded with the help of a digital camera. Plotting of ∆ absorbance vs. concentration of VIN was used to get a linear calibration plot in the resulting range.

### 2.5. Sensing of VIN in Real Water Samples with Clove-AgNPs

To detect VIN in real water samples, various blank water samples were collected from different areas of Turkey, mixed with the prepared AgNP solution, and then spiked with several concentrations of the VIN solution, according to the procedure used for standard solutions. The measurements were performed in triplicate each time, and the average concentration was taken as the actual concentration of the analyte in the sample.

## 3. Results and Discussion

### 3.1. UV-Vis Spectroscopy of Clove Extract-Derived AgNPs

Various studies have reported that the colorimetric behavior of nanoparticles depends on the size and geometry of the nanoparticles; thus, the optical response can be enhanced by reducing the dimensions of the nanoparticles [29,30]. The surface plasmon resonance behaviors of nanoparticles of metals such as Ag, Cu, and Au exist within the colorimetric region of the electronic spectrum [19,20,21,31,32]. UV-Vis spectroscopy was used as a preliminary tool to confirm the formation of clove extract-derived AgNPs. Optimization of various reaction parameters such as the volume of clove extract, amount of AgNO_3_, pH, and temperature was carried out in order to obtain stable, biosynthesized, AgNPs. A suitable amount of the precursor salt (0.1 M AgNO_3_) was used to obtain small AgNPs. The UV-Vis spectra presented in Figure S1 show an increase in signal response with a red shifted LSPR band with increasing quantity of Ag (0.1–0.7 mL of 0.1 M AgNO_3_). This difference in the LSPR band is attributed to aggregation phenomena with freely available Ag in solution. With a further increase in the amount of Ag (>0.7 mL), the LSPR band disappears and precipitation occurs. The solution becomes darker with increasing quantity of the salt, as depicted in the photograph in Figure S1.

The LSPR profile of clove extract solution (0.1–1.0 mL) indicates the reduction in particle size on the basis of the blue shifted UV-Vis spectral band as shown in Figure S2. An increase in quantity of clove extract results in the reduction of LSPR band intensity with a red shifted broadened peak. On the basis of the blue shifted LSPR band, the optimum amount of clove extract to obtain small and stable nanoparticles for further studies was determined to be 0.1 mL. Although there is no prominent change in the color, as shown in Figure S2, from the decrease in the intensity and blue shift of the LSPR band, the optimum amount of clove extract is determined to be 0.1 mL. pH is an important factor that affects the morphology and geometry of the nanoparticles. Therefore, in this study, the pH was optimized by varying it in the range of 4–12. The results show that in the acidic pH range, the nanoparticles easily oxidize because of interference by H^+^ ions, and therefore, an acidic pH does not favor the formation of nanoparticles. On the other hand, the spectral profile shows that a neutral pH (pH 7) is suitable for the formation of biosynthesized AgNPs (see Figure S3). The LSPR band of AgNPs synthesized at pH 7 shows a blue shift. Furthermore, the reaction temperature was optimized; the results (Figure S4) indicate that room temperature is suitable for the synthesis of AgNPs.

**Figure 1:**
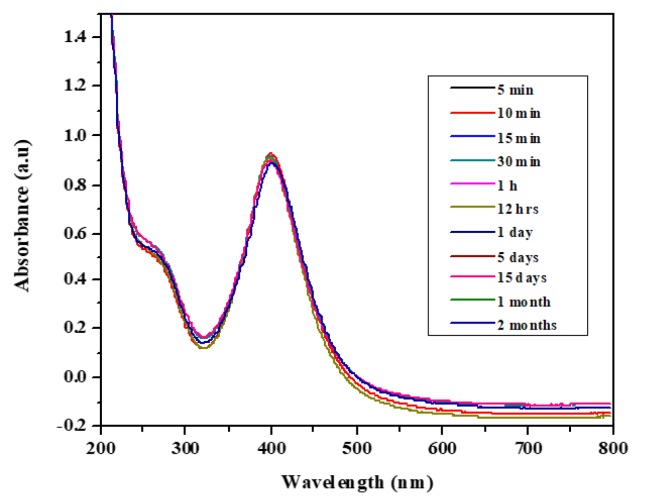
UV-Vis spectra of biosynthesized Ag nanoparticles (AgNPs) showing their stability.

Moreover, the stability of the biosynthesized AgNPs was studied as a function of time; the results show that the absorption wavelength (see Figure 1) and the color of the nanoparticle solution hardly changed in 2 months (at 397 nm). This indicates that the biologically synthesized AgNPs can be stored for a long time at room temperature and used any time for the detection of analytes.

### 3.2. FTIR and PXRD Analyses

**Figure 2:**
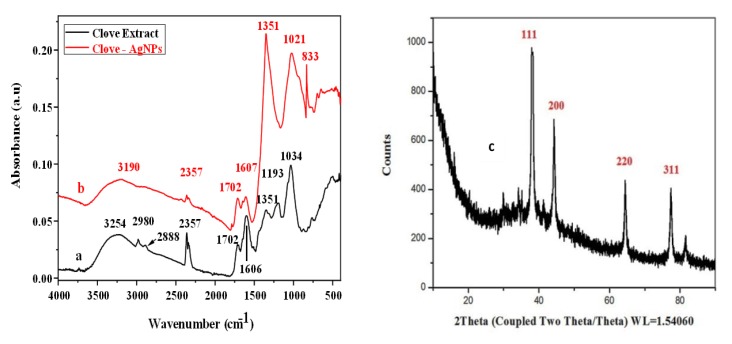
FTIR spectra of both clove extract (**a**) and clove-AgNPs (**b**) and (**c**) XRD patterns of biosynthesized AgNPs.

To study the interaction of clove extract with AgNPs and the crystalline nature of the AgNPs, FTIR spectroscopy and PXRD were performed (Figure 2). The FTIR results (Figure 2a,b) confirm the interaction of clove extract with AgNPs. Clove extract contains several organic compounds; eugenol is a major component, and it can act as a reducing agent, yielding nanoparticles. The peak at 3254 cm^−1^ in the FTIR spectrum of clove extract is attributed to the –OH group and the intense peak at 1034 cm^−1^ is attributed to the –CO functional group. In addition, the spectrum of clove extract contains various small peaks. As shown in Figure 2a, after the interaction of clove extract with AgNPs (Figure 2b), some bands disappear, and new bands appear. The appearance of a new broad, intense band at 1351 cm^−1^, which was absent in clove extract spectrum, indicates the formation of AgNPs and their interaction with reagents present in the clove extract. Signals at 2357 cm^−1^ (perhaps due to the possibility of CO_2_ contamination described at 2350 cm^−1^ [33]), 1702 cm^−1^, 1606 cm^−1^, and 1351 cm^−1^ in Figure 2a are repeated in Figure 2b but with changed absorbance due the interaction of the clove extract with AgNPs. The low signal at 1193 cm^−1^ in Figure 2a is absent due to intensification of peak at 1351 cm^−1^ in Figure 2b perhaps from overlapping.

In addition, compared to the –OH peak for clove extract, that for clove-AgNPs shifts towards a lower wavenumber (i.e., from 3254 cm^−1^ to 3190 cm^−1^), which is attributed to the interaction between clove extract and AgNPs through the OH linkage. Similarly, the –CO (carbon oxygen single bond) peak for clove-AgNPs shifts towards a lower wavenumber (i.e., from 1034 cm^−1^ to 1021 cm^−1^), further indicating the interaction of clove extract with AgNPs. The best verification of the interaction of AgNPs with the capping agent –OAg is the signal at 833 cm^−1^ in Figure 2b, which is absent in Figure 2a, because as cited elsewhere [34] the metal-O (metal Ag or Cu) appears at 834 cm^−1^. As a result of all these observations, it is confirmed that the AgNPs interacted with the capping agent.

Figure 2c presents the XRD crystalline patterns of clove-AgNPs. The patterns show four prominent peaks at 2θ values of 78, 63, 44, and 38°, which are assigned to the (311), (220), (200), and (111) planes of face centered cubic (fcc) crystal structure of clove-AgNPs [35].

### 3.3. Colorimetric Sensing of VIN by Aggregation-Based Mechanism (TEM Imaging)

**Figure 3:**
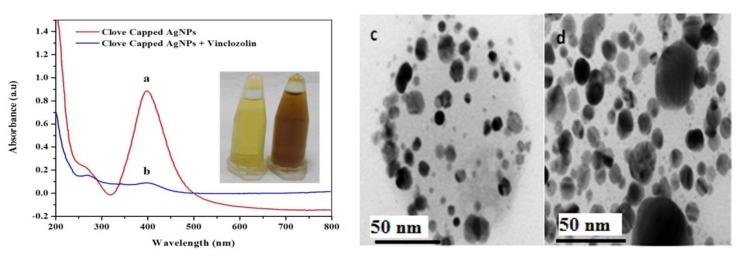
UV-Vis spectra of (**a**) clove-AgNPs and (**b**) clove-AgNPs + VIN (16 µM).(**c**) and (**d**) are the corresponding UHRTEM images for (**a**) and (**b**), respectively. Size range = 2–20 nm and average size = 14.4 nm of AgNPs (**c**).

For colorimetric detection of VIN, UV-Vis spectroscopy was performed. The UV-Vis spectra of clove extract-capped AgNPs before and after interaction with VIN are shown in Figure 3a,b, respectively.

As previously reported, the clove-AgNPs exhibited an LSPR band in the wavelength range of 380–450 nm [36]. However, after the addition of VIN (16 µM), the LSPR band intensity significantly decreases and the color of the AgNP solution changes from yellow to dark brown, as shown in the inset in Figure 3. Moreover, the addition of VIN leads to the formation of aggregates, as is evident from Figure 3c,d. Compared to the AgNPs after interaction with VIN (Figure 3b,d), those before the interaction (Figure 3a,c) are smaller because of the presence of capping molecules present in the clove extract; after VIN addition, AgNPs form aggregates, as indicated by a decrease in the absorbance and red shift in the absorption peak.

**Figure 4:**
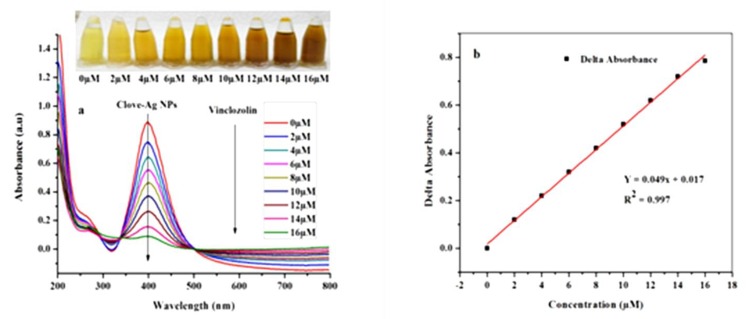
(**a**) UV-Vis absorbance spectra of AgNPs after addition of VIN (0–16 µM) and (**b**) the calibration curve: concentration of VIN vs. ∆ absorbance.

**Figure 5:**
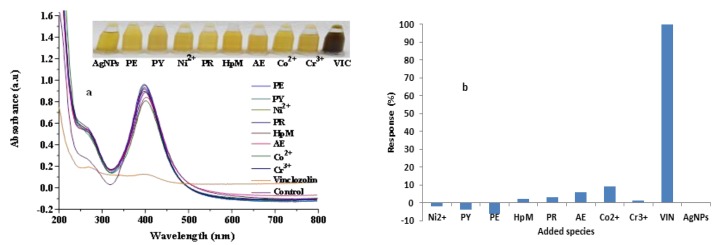
Effect of the addition of possible interfering agents as compared to that of VIN into AgNPs solution; (**a**) showing SPR spectra of AgNPs and interfering agents (inset showing corresponding photographs and (**b**) illustrating the corresponding bar graph.

**Scheme 2:**
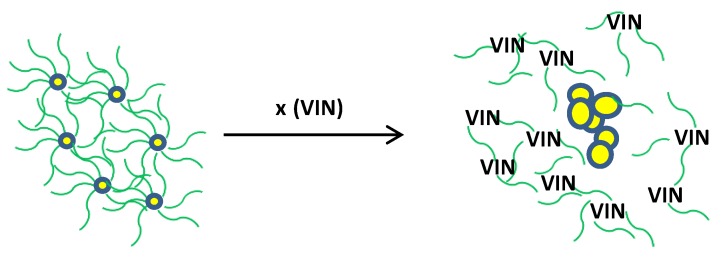
Sensing mechanism based on VIN-induced aggregation of AgNPs.

According to results obtained via UV-Vis spectrometry (Figure 3a,b) and UHRTM images (Figure 3c,d) it is evident that VIN is responsible for a decrease in the LSPR band of clove-based AgNPs as a result of removing the capping molecules from its surface to convert them into aggregated AgNPs (Figure 3b,d). The formation of dispersed AgNPs into aggregated AgNPs via VIN addition gives rise to the development of a colorimetric VIN sensor. Further support to these arguments could be provided via Figure 4 and Figure 5, where the VIN addition demonstrated the concentration dependent and selectivity-based sensing capability of the AgNPs solution via the aggregation of AgNPs resulting from the removal of coated or capped molecules adhered from the clove extract. A similar strategy has been discussed in other research for development of a Hg^2+^ sensor via Cu nanoparticles [37]. In view of this discussion, we propose the following mechanism for VIN sensing via clove-based AgNPs as depicted in Scheme 2.

In Scheme 2, 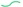 represents clove extract coating, 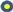 is an individual AgNP, the bigger circles are aggregated AgNPs formed after the addition of VIN, and x represents numerous molecules.

### 3.4. Calibration Study

The calibration curve, which shows the change in the absorbance with VIN concentration, was obtained in the range of 0–16 µM using a UV-Vis spectrophotometer (Figure 4b). The photographs of the corresponding solutions are shown in Figure 4. As observed, the color of the solution becomes increasingly dark with increasing concentration of VIN solution until it reaches the maximum value.

The limit of detection (LOD) and limit of quantification (LOQ) of the developed VIN colorimetric sensor were calculated from the standard deviation of the blank values (ð) and the slope of the linear plot: (3 × ð)/slope and (10 × ð)/slope, respectively [37]. Accordingly, the LOD and LOQ are 21 nM and 70 nM, respectively.

### 3.5. Selectivity of the Sensor

The selectivity of the clove-AgNP-based sensor toward VIN was studied by adding different metal salts (CrCl_3_, Ni(NO_3_)_2_, and Co(NO_3_)_2_) and pesticides (PY, PE, PR, HpM, LC, and AE) with a concentration of 160 µM as a 10:1 ratio to that of VIN to clove-AgNP solution. The inset photograph in Figure 5a describes the selective detection of VIN by AgNPs, as indicated by the abrupt color change after addition of VIN, compared to that after addition of other analytes (pesticides and metal ions). The increasing value of ∆ absorbance corresponds to a negative interference, while decreasing ∆ absorbance represents a positive interference. Notably, the color abruptly changes from yellow to dark brown after VIN addition, while the influence of other pesticides and metal ions on the LSPR band of clove-AgNPs is not prominent, as shown in Figure 5a. This result confirms that the fabricated sensor is highly selective and can easily detect low concentrations of VIN in the presence of other interferents.

The corresponding histogram (Figure 5b) shows the response levels of various pesticides and metal ions compared to that of VIN after addition into the AgNP solution. Thus, these results confirm the selectivity of the developed sensor.

### 3.6. Figure of Merit of the Developed VIN Sensor

Table 1 compares previously reported methods used for VIN detection with the AgNP sensor developed in this study.

It is clear from Table 1 that all methods are sensitive, except for that reported in [6]; however, they have the following disadvantages: high cost, complicated protocol, and/or lack of selectivity. Notably, our method is sensitive and novel, and this is the first report on VIN detection by UV-Vis spectrometry using clove extract-derived AgNPs. Moreover, this newly developed method or sensor is simple, rapid, and highly selective. All these properties distinguish the currently developed VIN sensing method from the others listed in Table 1.

### 3.7. Analysis of VIN in Real Water Samples

Analysis of real water samples were carried out to check the applicability of the developed sensor. Four real water samples were taken from different water resources in Turkey and the analyzed data are displayed in Table 2.

This table shows that the percentage recovery of VIN from real water samples (98.7–103) lies within the working range of the fabricated sensor, confirming that the fabricated sensor is suitable for VIN detection in the presence of other analytes and can be applied to other aqueous systems.

## 4. Conclusions

In this work, a green synthetic protocol for fabrication of biologically assisted, highly stable AgNPs using clove extract was developed, without using any chemical products. Fabricated AgNPs were characterized via several characterization techniques. UV-Vis and FTIR spectroscopy provided evidence regarding the LSPR and interaction of AgNPs with the capping agent, respectively. UHRTEM exhibited observations about size, shape and distribution while XRD defined the crystallinity of NPs. According to this characterization, the AgNPs showed a stable LSPR band at 397 nm, via interaction with the capping material as Ag(NPs)O-, with spherical NPs with an average size of around 14 nm having several crystalline planes at several 2θ angle values. The application study showed that the clove-AgNPs are highly selective and extremely sensitive candidates for trace level aggregation-based colorimetric sensing of endocrine disrupter fungicide, VIN with lower detection limit (LDL) of 21 nM, even in the presence of 10 times the amount of potentially interfering ions and molecules. The developed sensor was applied for testing of VIN in some real water. The sensor is sensitive, stable, facile, cost effective, selective, and quick in response which may beneficial for the poor people of society. The linear working range of the developed sensor works well above and below the permissible limit of VIN in water samples.

## Figures and Tables

**Table 1:** Comparison of reported methods with the colorimetric method developed in this study.

Method	LOD	Remarks	Reference
GC–MS/MS	0.5 ppb	Sensitive, but costly and complicated	[5]
HPLC	0.745 µM	Low sensitivity; high chemical consumption	[6]
SPME–GC–ECD	11.0 ppb	Sensitive, but costly and complicated	[7]
SPME–HPLC	4.0 ppb	Sensitive, slow, and complicated	[8]
GC–MS/MS	0.5 ppb	Sensitive, but costly and complicated	[9]
Electrochemical	11 nM	Sensitive; selectivity not studied	[10]
UV-Vis Spectrometry based on AgNP sol	21 nM	Sensitive, simple, and novel; reported for the first time	This work

GC-MS/MS; Gas chromatography tandem mass spectroscopy, HPLC; High performance liquid chromatography, SPME-GC-ECD; Solid phase micro-extraction coupled to GC with an electron capture detector, UV-Vis; Ultraviolet visible.

**Table 2:** Detection of VIN in various water samples using the developed sensor by the recovery method.

Water Sample from Turkey	VIN Added (µM)	VIN Found (µM)	Recovery (%)
Mediterranean Sea	05	05.10 ± 0.02	102.0
Ilgin Lake	10	10.30 ± 0.11	103.0
City Centre Konya	15	14.80 ± 0.15	098.7
Selcuk University Konya	15	14.90 ± 0.21	099.3

± Standard deviation of three replicates.

## Supplementary Materials

The following are available online at https://www.mdpi.com/2079-4991/9/11/1604/s1, Figure S1: Optimization of 0.1 M AgNO3, Figure S2: Optimization of volume of clove extract, Figure S3: pH study of AgNPs sol, Figure S4. Temperature effect on the formation of AgNPs.
