# Supplementary material for: Biogenic Silver Nanoparticles for Trace Colorimetric Sensing of Enzyme Disrupter Fungicide Vinclozolin
Source: Nanomaterials (Basel). 2019 Nov 12;9(11):1604. doi: 10.3390/nano9111604 (PMC6915375; doi:10.3390/nano9111604)
Supplement: Supplementary file 1 [file nanomaterials-09-01604-s001.pdf]

Supplementary Materials

Biogenic Silver Nanoparticles for Trace Colorimetric Sensing of Enzyme Disrupter Fungicide Vinclozolin

Masood Hussain [1], Ayman Nafady [2,3,*], Sirajuddin [4,*], Ahmet Avcı [5], Erol Pehlivan [6], Jan Nisar [7], Syed Tufail Hussain Sherazi [1], Aamna Balouch [1], Muhammad Raza Shah [4], Omar A. Almaghrabi [8] and Muhammad Anwar Ul-Haq [4]

[1] National Centre of Excellence in Analytical Chemistry, University of Sindh, Jamshoro 76080, Pakistan; masood.hussain59@yahoo.com (M.H.); tufail.sherazi@gmail.com (S.T.H.S.); aamna_balouch@yahoo.com (A.B.)
[2] Department of Chemistry, College of Science, King Saud University, Riyadh 11451 Saudi Arabia
[3] Chemistry Department, Faculty of Science, Sohag University, Sohag 82524, Egypt
[4] HEJ Research Institute of Chemistry, International Center for Chemical and Biological Center, University of Karachi, Karachi 75270, Pakistan; raza.shah@iccs.edu (M.R.S.); anwarulhaq196@gmail.com (A.U.)
[5] Department of Mechanical Engineering, Selcuk University, Konya 42079, Turkey; aavci@selcuk.edu.tr
[6] Department of Chemical Engineering, Selcuk University, Konya 42079, Turkey; erolpehlivan@gmail.com
[7] National Centre of Excellence in Physical Chemistry, University of Peshawar, Peshawar 25120, Pakistan; pashkalawati@gmail.com
[8] Department of Biological Sciences, Faculty of Science, University of Jeddah, Jeddah 21959, Saudi Arabia; oalmaghrabi@uj.edu.sa
* Correspondence: anafady@ksu.edu.sa (A.N.); drsiraj03@yahoo.com (S.)

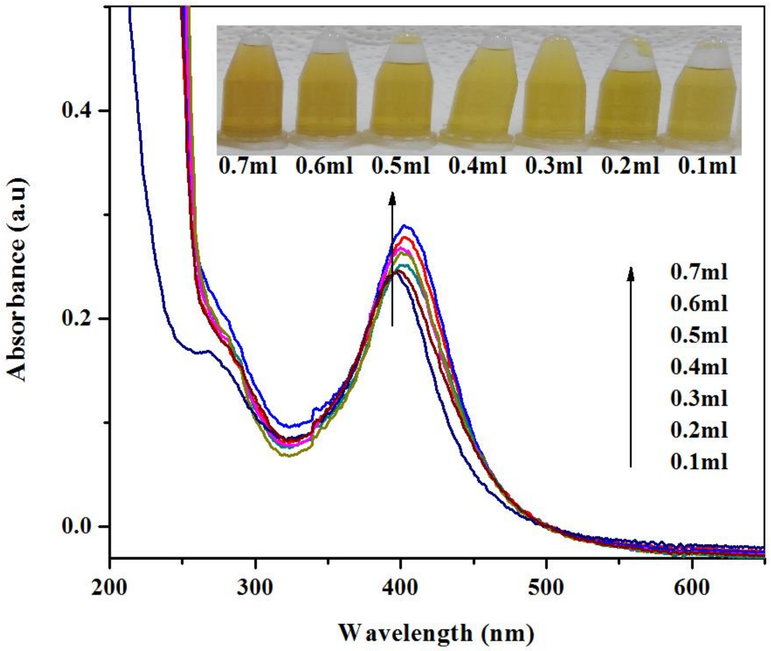

Figure S1. Optimization of 0.1 M $AgNO_3$.

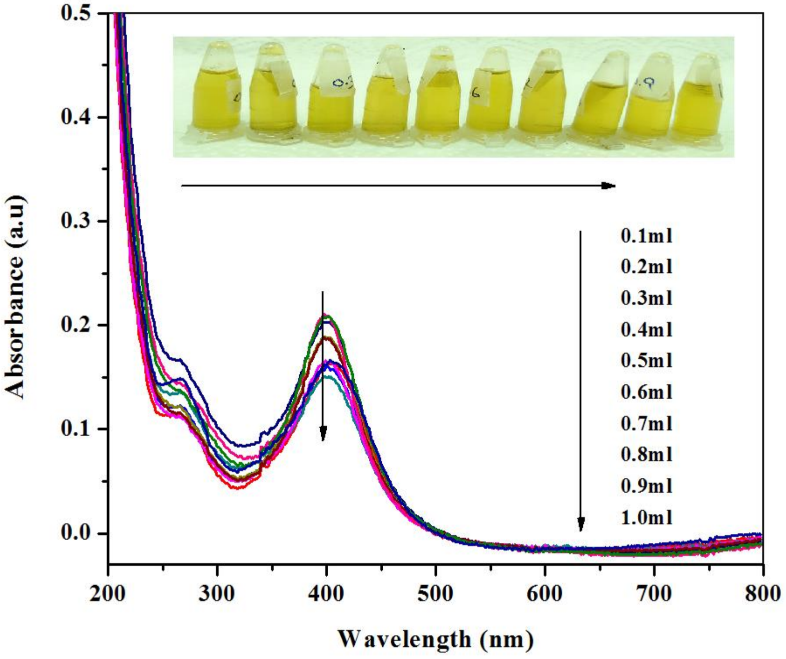

Figure S2. Optimization of volume of clove extract.

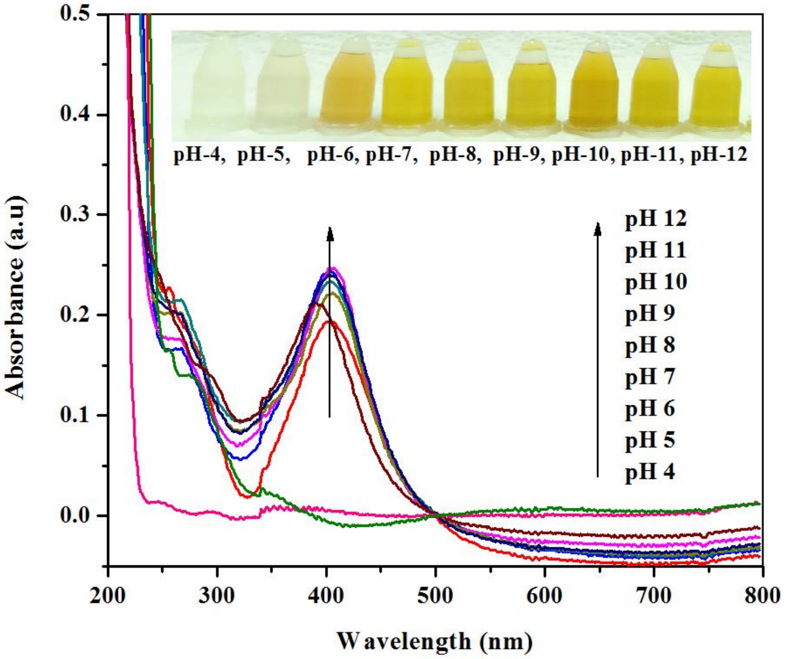

Figure S3. pH study of AgNPs sol.

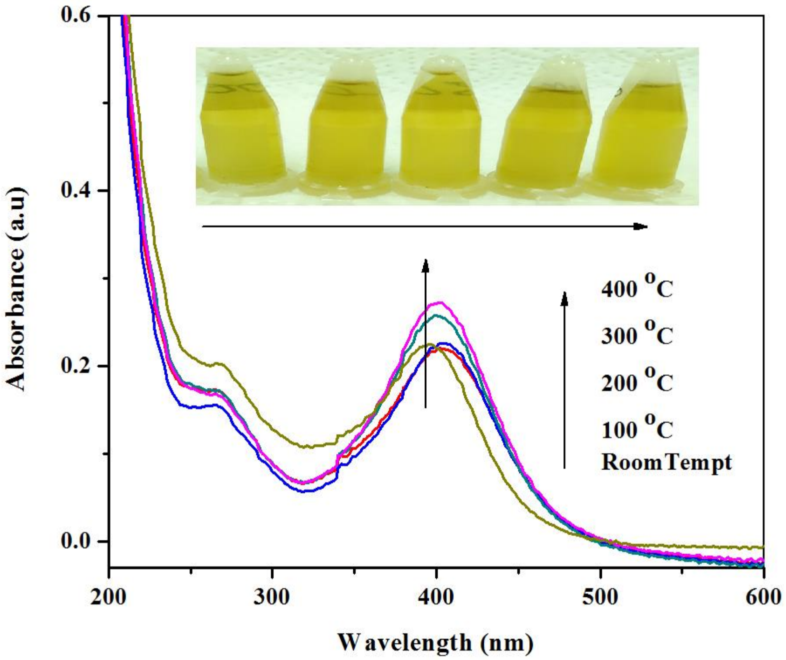

Figure S4. Temperature effect on the formation of AgNPs.
